# Anisotropic microtopography surface of chitosan scaffold regulating skin precursor-derived Schwann cells towards repair phenotype promotes neural regeneration

**DOI:** 10.1093/rb/rbae005

**Published:** 2024-01-27

**Authors:** Meng Cong, Xia Wu, Lingjie Zhu, Guohao Gu, Fei Ding, Guicai Li, Haiyan Shi

**Affiliations:** Key Laboratory of Neuroregenration of Jiangsu and Ministry of Education and Co-Innovation Center of Neuroregeneration, Nantong University, Nantong 226001, China; Key Laboratory of Neuroregenration of Jiangsu and Ministry of Education and Co-Innovation Center of Neuroregeneration, Nantong University, Nantong 226001, China; Department of Pathophysiology, School of Medicine, Nantong University, Nantong 226001, China; Key Laboratory of Neuroregenration of Jiangsu and Ministry of Education and Co-Innovation Center of Neuroregeneration, Nantong University, Nantong 226001, China; Key Laboratory of Neuroregenration of Jiangsu and Ministry of Education and Co-Innovation Center of Neuroregeneration, Nantong University, Nantong 226001, China; Key Laboratory of Neuroregenration of Jiangsu and Ministry of Education and Co-Innovation Center of Neuroregeneration, Nantong University, Nantong 226001, China; Key Laboratory of Neuroregenration of Jiangsu and Ministry of Education and Co-Innovation Center of Neuroregeneration, Nantong University, Nantong 226001, China; Department of Pathophysiology, School of Medicine, Nantong University, Nantong 226001, China

**Keywords:** anisotropic microtopography, skin precursor-derived Schwann cells, chitosan-film, neural regeneration, phenotype regulation

## Abstract

For repairing peripheral nerve and spinal cord defects, biomaterial scaffold-based cell-therapy was emerged as an effective strategy, requiring the positive response of seed cells to biomaterial substrate and environment signals. Previous work highlighted that the imposed surface properties of scaffold could provide important guidance cues to adhered cells for polarization. However, the insufficiency of native Schwann cells and unclear cellular response mechanisms remained to be addressed. Given that, this study aimed to illuminate the micropatterned chitosan-film action on the rat skin precursor-derived Schwann cells (SKP-SCs). Chitosan-film with different ridge/groove size was fabricated and applied for the SKP-SCs induction. Results indicated that SKP-SCs cultured on 30 μm size microgroove surface showed better oriented alignment phenotype. Induced SKP-SCs presented similar genic phenotype as repair Schwann cells, increasing expression of c-Jun, neural cell adhesion molecule, and neurotrophic receptor p75. Moreover, SKP-SC-secretome was subjected to cytokine array GS67 assay, data indicated the regulation of paracrine phenotype, a panel of cytokines was verified up-regulated at secreted level and gene expression level in induced SKP-SCs. These up-regulated cytokines exhibit a series of promotive neural regeneration functions, including cell survival, cell migration, cell proliferation, angiogenesis, axon growth, and cellular organization etc. through bioinformatics analysis. Furthermore, the effectively polarized SKP-SCs-sourced secretome, promoted the proliferation and migration capacity of the primarily cultured native rat Schwann cells, and augmented neurites growth of the cultured motoneurons, as well as boosted axonal regrowth of the axotomy-injured motoneurons. Taken together, SKP-SCs obtained pro-neuroregeneration phenotype in adaptive response to the anisotropic topography surface of chitosan-film, displayed the oriented parallel growth, the transition towards repair Schwann cell genic phenotype, and the enhanced paracrine effect on neural regeneration. This study provided novel insights into the potency of anisotropic microtopography surface to Schwann-like cells phenotype regulation, that facilitating to provide promising engineered cell-scaffold in neural injury therapies.

## Graphical Abstract

**Figure:**
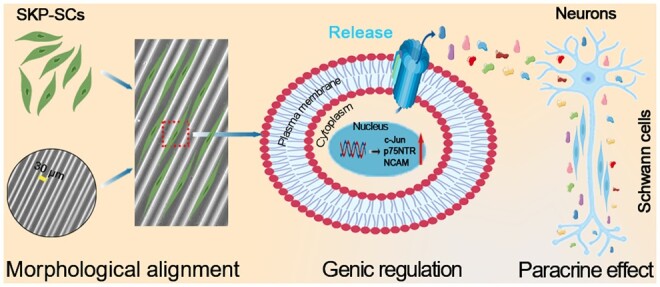

## Introduction

In neural regeneration medicine field, human still face the challenge on repairing spinal cord injury (SCI) and long-gap peripheral nerve injury (PNI), attributed to either the source shortage and size mismatch of gold standard nerve autografts for PNI [1, 2], and the remained dissatisfactory surgery and pharmaceutical treatment efficacy for SCI [3, 4]. The irreversible sensory and locomotive disability of patients, and a significant socioeconomic burden on global public health business, urging for exploring more reasonable and feasible strategies [5, 6]. The research on the molecular mechanisms of nerve injury, microsurgical techniques, and stem cell research have made substantial progress in the field of translational neurobiology. The current research on peripheral nerve regeneration aims to accelerate its potential use through stem cells and extracellular vesicles (EVs), drug formulations, and neural conduit bioengineering [7]. Among these strategies, cell and tissue regenerative engineering approaches have been in rapid development [8, 9].

Researchers have introduced critical elements to develop neural tissue regenerative engineering. For instance, the biomaterial scaffold constructs enable the nerve stumps to form new gap-bridge guiding axon regrowth [9], and the implantation of stem cells and derivative cells can make up the shortage of native tissue cells [10], while cell-free therapy with biophysical and biochemical stimuli might avoid immune rejection and ethics issues [11]. Especially, the combinational options, such as scaffolds loaded with cells and/or bioactive agents demonstrated significant improvement of functional recovery compared to empty scaffolds, the engineered tissue module can induce dorsal root ganglion and spinal cord neurons remodeling axonal continuity [12, 13]. Given that, the utilization of interdisciplinary technologies has been evidenced to be indispensable and prospect strategies for regeneration of peripheral nerve fibers and spinal cord neurite tracts.

Since biomaterial scaffolds served as the primary component in tissue engineering, recent decades plenty of biomaterials have been developed. Various tissue-engineered grafts with scaffold fabricated by natural or synthetic materials have been applied to bridge nerve gap and speed up neural regeneration [14, 15]. Recently, the electroactive biomaterials and systems for cell fate determination and tissue regeneration was designed and applied [16]. Besides, a bioinspired injectable, adhesive, and self-healing hydrogel with dual hybrid network for neural regeneration after SCI was fabricated and applied [17]. It is noteworthy that chitosan substrate has been proved as one of the biocompatible and biodegradable natural polymers with sufficient biomechanics and suitable porosity [18], and has been approved by the Food and Drug Administration for medical use [19]. The plain chitosan conduits have been evidenced to be applicable for SCI repair in fundamental research strategy [20], and approved for clinical use in Europe [21]. Chitosan has also shown good performances for the fabrication of neural conduits combining with various bioactive molecules owing to the polycation characteristics [18]. Meanwhile, continuous development on surface feature is also required to chitosan scaffold fabrication, particularly, an imposed anisotropic microstructure property beneficial for peripheral nerve and spinal cord regeneration attract much attention [22]. Various kinds of approaches have been developed to obtain micropatterned surface. Our previous work has successfully constructed anisotropic topographic chitosan membrane with parallel ridge/groove utilizing the stamp-molding technique, that simple approach is applicable for chitosan polymer, and the microtopographic surface cues of scaffold is convenient to guiding cell polarization [23, 24].

Additionally, the seed cell is an essential element in tissue engineering to replenish native tissue cells at the lesion location. The peripheral nervous glial cell type, Schwann cells (SCs), was well known to repair mild PNI instinctively, that has motivated the application of SCs transplantation in therapy studies. Corresponding works have achieved a series of positive outcome on both peripheral and central nerve injury repair improvement [25, 26]. Nevertheless, the insufficiency of native SCs hindered the practical translational application. Thus researchers have attempted to utilize Schwann-like cells derived from distinct stem cells/precursors to substitute native SCs, including bone marrow mesenchymal stem cells, adipose stem cells, skin-derived precursory cells, pluripotent stem cells, etc [27]. Among them, the advantage of SCs derived from skin precursors (SKP-SCs) was outstanding due to their identical neural crest cell developmental origin with native SCs and high purity [28]. The feasibility of utilizing SKP-SCs was not only supported by their practical availability, but also their therapeutic effectiveness on PNI and SCI [29, 30]. Moreover, it has been recognized that the seed cells on scaffolds could exert further pro-regeneration effect through paracrine signals [31], comprising soluble bioactive molecules, extracellular matrix (ECM) components, and EVs-sourced cargoes etc. [11], namely cell secretome.

Based on previous findings, we anticipate that the combination of SKP-SCs with anisotropic chitosan scaffold may be beneficial for fostering neural regeneration. In present work, we focused on the influence of different size microgroove patterned surface of chitosan film on SKP-SCs polarization, including morphology phenotype transition and genic phenotype reprogramming, as well as paracrine phenotype regulation. Meanwhile, the adaptive response of SKP-SCs to anisotropic topography were uncovered. Moreover, the secretome from polarized SKP-SCs was further detected and informatively analysed to finding potent chemotaxis and trophic cytokines. Then SKP-SCs secretome was utilized to treat native SCs and damaged primary motoneurons (MNs) to evaluate the paracrine pro-neuroregenerative function of topography-induced SKP-SCs. The findings would provide experimental evidences to further study on the application of Schwann-like cells combining with anisotropic biomaterial scaffold.

## Materials and methods

### Animals

Sprague Dawley (SD) rats (male) and green fluorescence protein (GFP)-positive transgenic male SD rats (male) were provided by the Laboratory Animal Services Centre of Nantong University. All the animal experimental procedures were approved by the Jiangsu Provincial Administration Committee of Experimental Animals (No. 20150305-030) according the Institutional Animal Care Guidelines of Nantong University, and ethically approved by the Laboratory Animal Center of Nantong University (No. IACUC202202151005, February 15, 2022).

### Rat primary SKPs culture and differentiation toward SCs

**Scheme 1.**
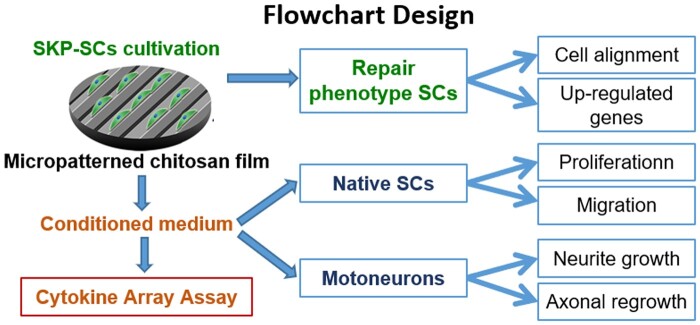
A flowchart design of experiments in this study.

Rat SKP-SCs were isolated and cultured as previously described [32, 33]. Briefly, back skin tissue was dissected from newborn rats and digested with collagenase type XI (Sigma, St Louis, MO, USA). The collected single cell suspension was plated at a density of 3 × 10^4^ cells/ml in proliferation medium containing DMEM/F-12 (v/v: 3:1, Corning, Manassas, VA, USA), 20 ng/ml epidermal growth factor (EGF, R&D, Minneapolis, MN, USA), 40 ng/ml basic fibroblast growth factor (bFGF, R&D), 2% B27 supplement (Gibco, Life Technology, NY, USA), and 1% antibiotics (Gibco). Primary skin precursors (SKPs) culture was passaged every 12–14 days. Post-three-passage, were induced to differentiate toward SCs in 5 × 10^4^ cells/ml inoculating concentration on Poly-D-lysine (PDL, Sigma)/laminin (Sigma) coated plates, with medium containing DMEM/F-12 (v/v: 3:1) (Corning), 50 ng/ml Heregulin-1β (R&D), 2% N2 supplement (Stem Cell Technologies, Vancouver, BC, Canada), 5 μM forskolin (Sigma), 3% fetal bovine serum (Sigma) and 1% penicillin/streptomycin (Beyotime, Shanghai, China). Usually, SC colonies were obtained after 2–3 weeks, SKP-SCs were cryopreserved at early passages for subsequent experimental application. The cryopreserved SKP-SCs were subjected to resuscitation and expansion culture according to demand. In consistence with our previous work, three continuous generations of SKP-SCs from passage 14 (P14) to P16 were used in our work for conditioned media collection and treatment studies. The flowchart of key steps is shown in Scheme 1.

### Immunofluorescence staining

GFP-positive SKP-SCs were fixed with 4% paraformaldehyde, and blocked, then incubated with rabbit anti-S100-β primary antibody (1:400, ThermoFisher Scientific, Carlsbad, CA, USA) overnight at 4°C, followed by reaction with goat anti-rabbit-IgM-cyanine 3 (Cy3) (1:400, Abcam, Cambridge, UK) and Hoechst 33342 (Abcam) counterstaining. The cell samples were observed under a Confocal Laser Scanning Microscope (TCS SP5, Leica, Mannheim, Germany).

### Fabrication of micropatterned chitosan film

The micropatterned chitosan film was prepared as previously described [34]. First, the polydimethylsiloxane (PDMS, Corning) stamp was prepared with a 2D structure consisting of parallel channels and ridges. The micropatterned silicon panels were washed by ultrapure water. Then, thoroughly mix the PDMS solution with the fixative in a ratio of 10:1 and remove bubbles under vacuum at room temperature. Subsequently, PDMS solution was poured onto the silicon panels and cured at 80°C in a vacuum oven for 2 h. Then, peeled off from silicon panels to obtain PDMS stamp. Three sizes of PDMS stamp with different ridge/groove dimension were prepared, including10/10 μm, 30/30 μm and 50/50 μm width with the same depth (10 μm). Finally, the chitosan powder (Xincheng Biochemical Company, Nantong, China; Mw: 2.8 × 10^4^; degree of deacetylation: 92.3%.) was dissolved in a 1 wt.% acetic acid aqueous solution to form a 1 wt.% chitosan solution at room temperature, then stewing 2 h to remove the trapped air bubbles. The pH value of the solution was in the range of 4.0–5.0. Afterwards, a 100 μl of 1 wt% chitosan solution was dropped onto each coverslip, and the PDMS stamps were pressed on coverslips for up to 48 h at room temperature. After drying, the PDMS stamps were removed, thus forming the micropatterned chitosan films. The chitosan films without micropattern (i.e. flat film) were prepared as control. In previous work, the stability of micropatterned chitosan films has been examined by immersing the samples into PBS for 12 d, indicating a better stability of the porous chitosan micropatterning with large size was more than the sample with small micropatterning size [23].

### Observation of morphology of micropatterned chitosan films

The morphology of micropatterned chitosan films was observed under scanning electron microscopy (SEM, Hitachi S-3400, Tokyo, Japan). Briefly, a double-sided adhesive tape was used to fix the samples to an aluminum platform, then the samples were coated with a layer of gold (about 50 nm). Finally, the coated samples were observed under SEM by the vacuum degree of 1.33 × 10^−4 ^Pa at an acceleration voltage of 20 kV.

### SKP-SCs cultivation on chitosan films

For culturing SKP-SCs on the substrate surface, the flat and micropatterned chitosan films were rinsing with phosphate buffer solution (PBS) twice. Then the films were transferred to a 24-well plate and coated with PDL/laminin for 2 h at 37°C, followed with washing with PBS and sterilizing by ultraviolet light for 2 h. Finally, 1 ml cell suspension with a concentration of 4 × 10^4^ cells/ml was seeded onto the films in each well.

### Observation of micromorphology of SKP-SCs

For micromorphology observation, all samples were firstly fixed with 25% glutaraldehyde for 2 h at 4°C, and thereafter stained with for osmium acid for 2 h at room temperature. Then samples were successively dehydrated for 15 min in each grade, at increasing alcohol concentrations (50%, 70%, 80%, 95% and 100%; V-alcohol/V-demineralized water), next dried at room temperature for more than 24 h. Finally, the micromorphology of SKP-SCs was observed under SEM after coating with gold.

### Morphometric analysis of SKP-SCs

GFP-positive SKP-SCs cultured on different size microgroove surface of chitosan film samples were observed under an Inverted Fluorescence Microscope (IX71, Olympus, Tokyo, Japan) to obtain cell images. The morphological indexes of SKP-SCs, including the cell area, the cell aspect ratio, and the cell angle, were measured according to the method described previously [35]. The cell area index was to describe the cell spreading extent on the micropatterned surface. The cell aspect ratio was to indicate the elongation shape of cells, generally, the bigger the ratio of length/width the more obvious the cell elongation. The cell angle was defined as the angle between the long axis of the cell and the microgroove direction, be used to describe whether the cell orientation was consistent with the topographic direction. All the indexes of SKP-SCs on micropatterned and flat samples were detected at 12 h, 24 h and 36 h, respectively, next were measured by Image J software (version 1.8.0; National Institutes of Health, Bethesda, MD, USA). Four parallel samples were used for 10 μm, 30 μm, 50 μm size microgroove surface groups and flat surface group, respectively. For each sample, the fluorescence microscope (IX71, Olympus) was used to image five randomly chosen fields, and then eight to ten cells were analysed from every image to obtain a better statistical assessment of the morphological indexes.

### Conditioned medium collection for cell counting assay of SKP-SCs

The SKP-SCs conditioned medium (CM) was obtained by culturing SKP-SCs on 30 μm micropatterned chitosan film (indicated as ‘30 μm’), and CM collected from flat film group (indicated as ‘flat’) was served as control. At 36 h after culture, medium was switched to serum-free medium for another 48 h. The cell culture supernatant, namely CM, also termed as secretome, was collected and underwent sequential 800 g ultracentrifugation for 10 min at 4°C to remove the cell debris. The collected CM were stored at minus 80 degree for subsequent experiments.

Cell counting kit-8 assay (CCK-8, Dojindo, Tokyo, Japan) was used to investigate the proliferation of SKP-SCs. In brief, after SKP-SCs were cultured for 36 h on 10 μm, 30 μm, 50 μm size microgroove surface groups and flat surface group, the medium was discarded and the samples were rinsed with PBS twice. Then, fresh medium containing CCK-8 reagent (v/v = 10:1) was added to each sample and incubated at 37°C for another 4 h. Afterwards, 100 μl of suspension was transferred to a 96-well plate with at least six repeated wells each group. The value of optical density (OD) was measured at 450 nm using a microplate reader (BioTek, Burlington, VT, USA).

### Quantitative reverse transcriptase-polymerase chain reaction

Total RNA was isolated from SKP-SCs using Trizol reagent (Qiagen, Valencia, CA, USA), and cDNA was obtained by Omniscript RT kit (Qiagen) according to the manufacturer’s instructions. Quantitative reverse transcriptase-polymerase chain reaction (qRT-PCR) was performed with SYBR Premix (Roche) on the BIO-RAD system (BIO-RAD-96CFX) according to standard methods. The specific method was based on the manufacturer’s instructions. The results were analysed by the 2^−△△Ct^ method. The primers for rat mRNA detection are listed in Supplementary Table S1.

### Cytokine array assay and bioinformatic analysis of SKP-SC-sourced secretome

On the basis of the best alignment morphology of SKP-SCs on ‘30 μm’ surface, SKP-SC-sourced secretome (indicated as ‘30 μm-CM’) was collected to compare with that from ‘flat’ surface (indicated as ‘flat-CM’). The cytokine arrays were tested following the manufacturer’s instructions of Rat Cytokine Array GS67 (RayBiotech, Guangzhou, China). In order to gain insight into the biological significance of differentially expressed proteins, the up- or down-regulated cytokines in ‘30 μm-CM’ were screened out according to the appropriate log_2_ fold change (log_2_FC) standard and −log10 (*P*-value) criteria. After further verification of the mRNA expression level of these up-regulated cytokines in SKP-SCs from ‘30 μm-CM’ group and ‘flat-CM’ group, the cluster candidate cytokines were further categorized via bioinformatic analysis based on the biological function annotations from the UniProt database (http://www.uniprot.org).

### Primary culture of SCs

The native SCs were isolated from sciatic nerves of 1- to 3-day-old SD rats, and purified to remove the fibroblasts using anti-Thy1.1 antibody (Sigma) and rabbit complement (ThermoFisher Scientific) as previously described [36]. The primary SCs with cell confluence of 95% was identified by immunostaining with S100-β, a specific SC marker.

### Conditioned medium concentration and cell proliferation assay of SCs

To obtain the 20 times concentrated supernatants, the SKP-SCs supernatants was added to the concentrated filter column (Amicon Ultra-15 Centrifugal Filter Devices, washing by sterilized Milli-Q water twice) and centrifuged with 3000 g for 20 min at 25°C. The concentrated CM would be added into primary cell culture medium at 10% concentration to treat neural cells *in vitro*.

The cell proliferation was analysed by a cell-light 5-ethynyl-2′-deoxyuridine (EdU) DNA cell proliferation kit (Ribobio, Guangdong, China) according to the manufacturer’s protocol. SCs were plated at a density of 1 × 10^5^ cells/ml onto poly-L-lysine (Sigma)-coated 96-well plates with triplicate wells in each group. At the indicated time points after cell treatment with ‘30 μm-CM’, ‘flat-CM’ or primary SCs culture medium (indicated as ‘SC-medium’), 50 mM EdU labeling medium was added into each well, then incubated for an additional 2 h. Finally, images were obtained with Inverted Fuorescence Microscope (Olympus) and analysed with Image J software. SC proliferation was expressed as the ratio of EdU-positive cell nuclei (red) to total cell nuclei (blue).

### Migration assay of SCs

The cell migration assays were performed using the culture-insert two well device (IBIDI, Fitchburg, WI, USA) according to the manufacturer’s protocol. After SCs attachment for 12 h, the culture-insert was gently removed with sterile tweezers, then SCs were treated with ‘30 μm-CM’, ‘flat-CM’ or ‘SC-medium’. Following culture for additional 8 h, the scratches in different groups were observed and images were captured with Inverted Microscope (Olympus), then images were analysed with Image J software to calculate the percentage of migration area.

### Primary culture of MNs and neurite growth detection

Primary MNs were obtained from SD rat embryos (13.5d) as previously described [37]. Briefly, spinal cords were isolated and digested with 0.125% trypsin for 30 min at 37°C, then 15% OptiPrep gradient centrifugation solution (Sigma) was used to purify MNs. Cells cultured on coverslips coated with Poly-lysine in neurobasal medium (Gibco) with 2% B27 (Gibco), 1% mM L-glutamine (Gibco), and 1% penicillin/streptomycin. The MNs purity was determined by immunostaining with chicken anti-choline acetyltransferase (ChAT) antibody (1:200, Abcam) and rabbit anti-neurofilament 200 (NF200) antibody (1:400, Sigma) at 4°C overnight, followed by incubation with goat anti rabbit IgG-Cy3 (1:400, Abcam) and donkey anti chicken IgG-fluorescein isothiocyanate (FITC) (1:400, Abcam) with final Hoechst 33342 counterstaining.

To evaluate the effect of SKP-SC-sourced secretome (indicated as ‘SKP-SC-secretome’) on the neurite growth of MNs, MNs were treated for 24 h with ‘30 μm-CM’, ‘flat-CM’, or primary MNs culture medium (indicated as ‘MN-medium’). Then the immunostaining for NF200 was performed, and the captured images were analysed by Image J software. Two parameters for MNs neurite growth detection were assessed in per hundred neurons, including the average length of the longest neurite, and the neurite density ratio, namely the ratio of branch numbers to cell numbers.

Besides, the axonal regrowth of MNs post-axotomy injury was further detected *in vitro* by the microfluidic device with 150 μm microgrooves (SND 150, Xona Microfluidics, Temecula, CA, USA) as previously described [38]. Briefly, microfluidic devices were attached to the poly-l-lysine-coated cover petri dish after ultraviolet irradiation sterilization, then 10 μl MNs suspension at 2 × 10^7^ cells/ml concentration culture media was loaded into the soma chamber of each microfluidic device. Then, the axon transection model of MNs was established after 4-day regular culture, the axonal compartment was aspirated twice for 1–2 min each time with ≥18 in-Hg vacuum pressure to ensure the axons were completely transected. Afterword, the ‘30 μm-CM’, ‘flat-CM’, and ‘MN-medium’ was added into chamber for 24 h, respectively. MNs could regrow out axons, after fixation the axons were incubated with mouse anti-β-tubulin3 (TUJ1) (1:400, Abcam) and sequential goat anti mouse IgG-FITC (1:400, Abcam). The images were taken under the Inverted Fluorescence Microscope (DMi8, Leica, Mannheim, Germany). For three groups, the average length of the 15 longest axons was assessed.

### Statistical analysis

All quantitative data were presented as mean ± standard error of the mean from at least three independent experiments. The statistical significance was analysed using GraphPad Prism 8.0.1 software (GraphPad Prism Software Inc, San Diego, CA, USA). Comparison between two groups was assessed by Paired T test, comparison among three or more groups was assessed by Ordinary one-way analysis of variance (ANOVA) Tukey’s test, and comparison of two variables among three or more groups was assessed by two-way ANOVA Tukey’s test. In all comparison, *P *<* *0.05 was considered statistically significant.

## Results

### Characterization of SKPs and SKP-SCs

**Figure 1.**
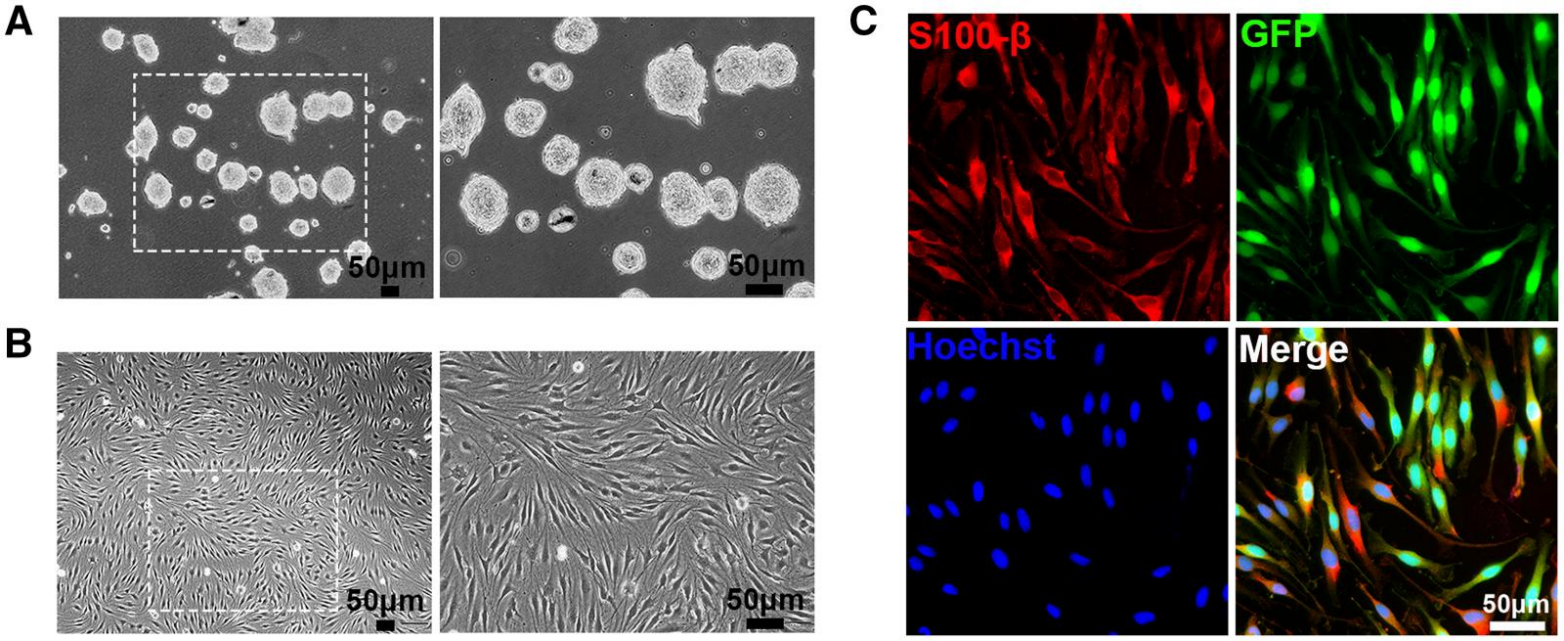
Characterization of SKPs and SKP-derived SCs. (**A**) Cultured SKPs showed floating spherical clone morphology. (**B**) SKP-derived SCs displayed long-spindle shape and side-by-side alignment. (**C**) GFP (green)-positive SKP-SCs showed expression of SC marker S100-β (red) with Hoechst (blue) labeled cell nuclei. Scale bar, 50 μm.

Primary cultured rat SKPs were self-renewing, always growing as floating spherical clones when expanded in culture medium and observed under phase-contrast microscope (Figure 1A). After committed induction of differentiation, obtained SKP-SCs displayed long-spindle shape with side-by-side alignment (Figure 1B), and GFP-positive SKP-SCs expressed SC-specific marker S100-β (Figure 1C). Results demonstrated that the SKP-SCs were available for our work.

### Morphology of micropatterned chitosan films and adhered SKP-SCs

**Figure 2.**
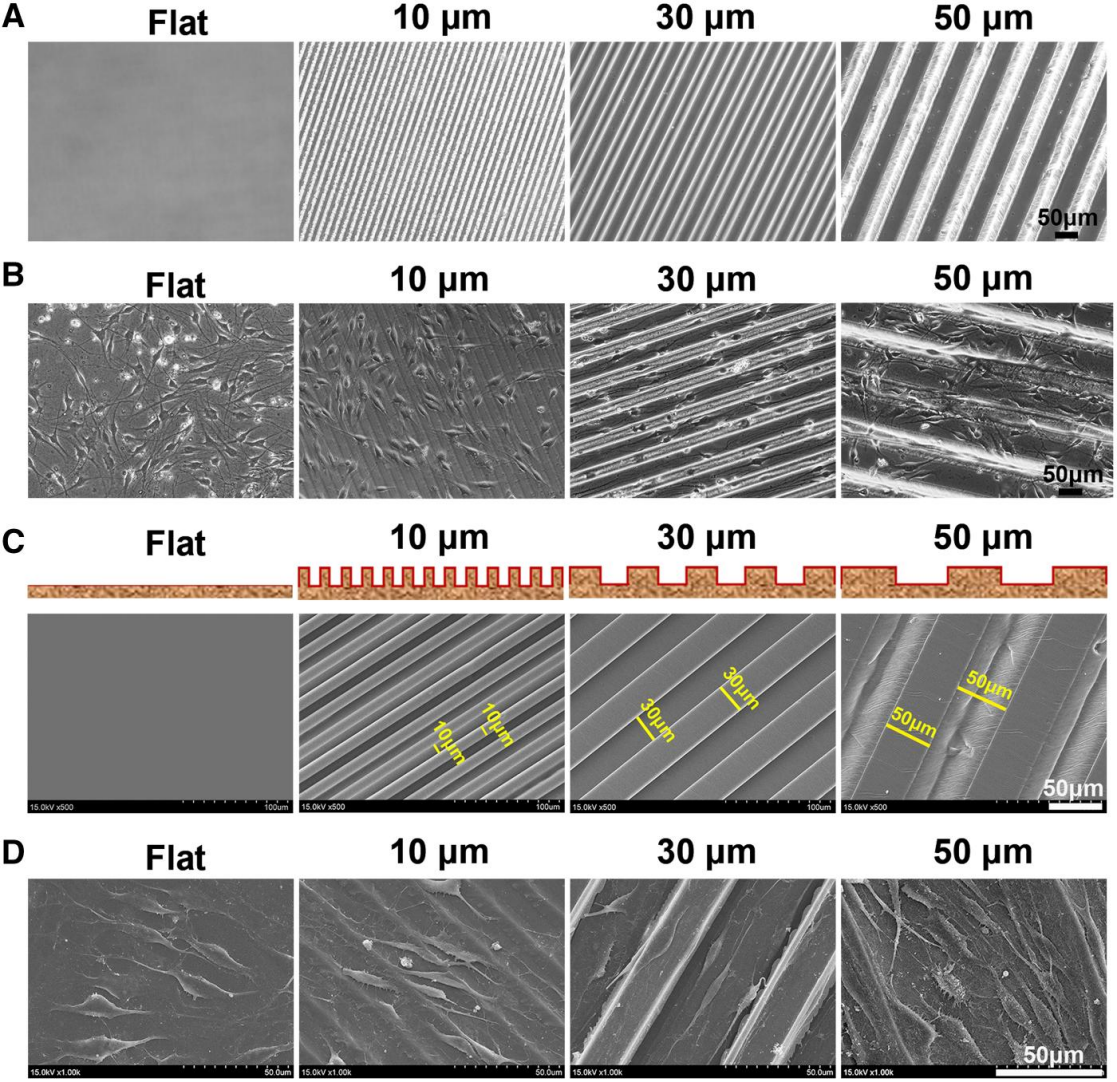
Optical images and SEM images of micropatterned chitosan films and adhered SKP-SCs. (**A**) Representative optical images of micropatterned chitosan films with different size of ridge/groove, flat film served as control. (**B**) Representative optical images of SKP-SCs cultured on different chitosan film surface groups for 36 h, showing the random arranged SKP-SCs on flat film surface obtained the regular arrangement on micropatterned film. (**C**) Representative SEM images of the microstructure of chitosan film in four groups, and the simulation maps of the cross-sections of different chitosan films. (**D**) Representative SEM images of adhered SKP-SCs showing best arrangement on chitosan film surface with 30 μm size of ridge/groove, and improved arrangement of SKP-SCs in ‘10 μm’ and ‘50 μm’ group than that in ‘flat’ group. Scale bar, 50 μm.

The optical images showed four types of chitosan film, the flat chitosan film group with the smooth surface was set as control, and three micropatterned film groups exhibited 10/10 μm, 30/30 μm and 50/50 μm size ordered ridge/groove, respectively (Figure 2A). After culture for 36 h, the adherent SKP-SCs cultured on micropatterned chitosan film surface showed relatively ordered alignment than unordered cells on flat film surface (Figure 2B). Similar as that shown in the optical images, under SEM the microstructure of bridge/groove was presented clearly, meanwhile, the simulation maps of the cross-sections of different chitosan films was exhibited (Figure 2C). Besides, SKP-SCs on ‘flat’ surface showed random distribution without any regular orientation, on the contrast, nearly all SKP-SCs arranged well along with the axial position of ridge/groove on the ‘30 μm’ surface, while on the ‘10 μm’ and ‘50 μm’ surface some SKP-SCs still showing non-axial position arrangement (Figure 2D). It suggested that SKP-SCs exhibited orderly arrangement and elongated morphology via topographic cues induction, and the ‘30 μm’ surface showed the advantage than the ‘10 μm’ and ‘50 μm’ surface.

### Morphological alignment of GFP-positive SKP-SCs

**Figure 3.**
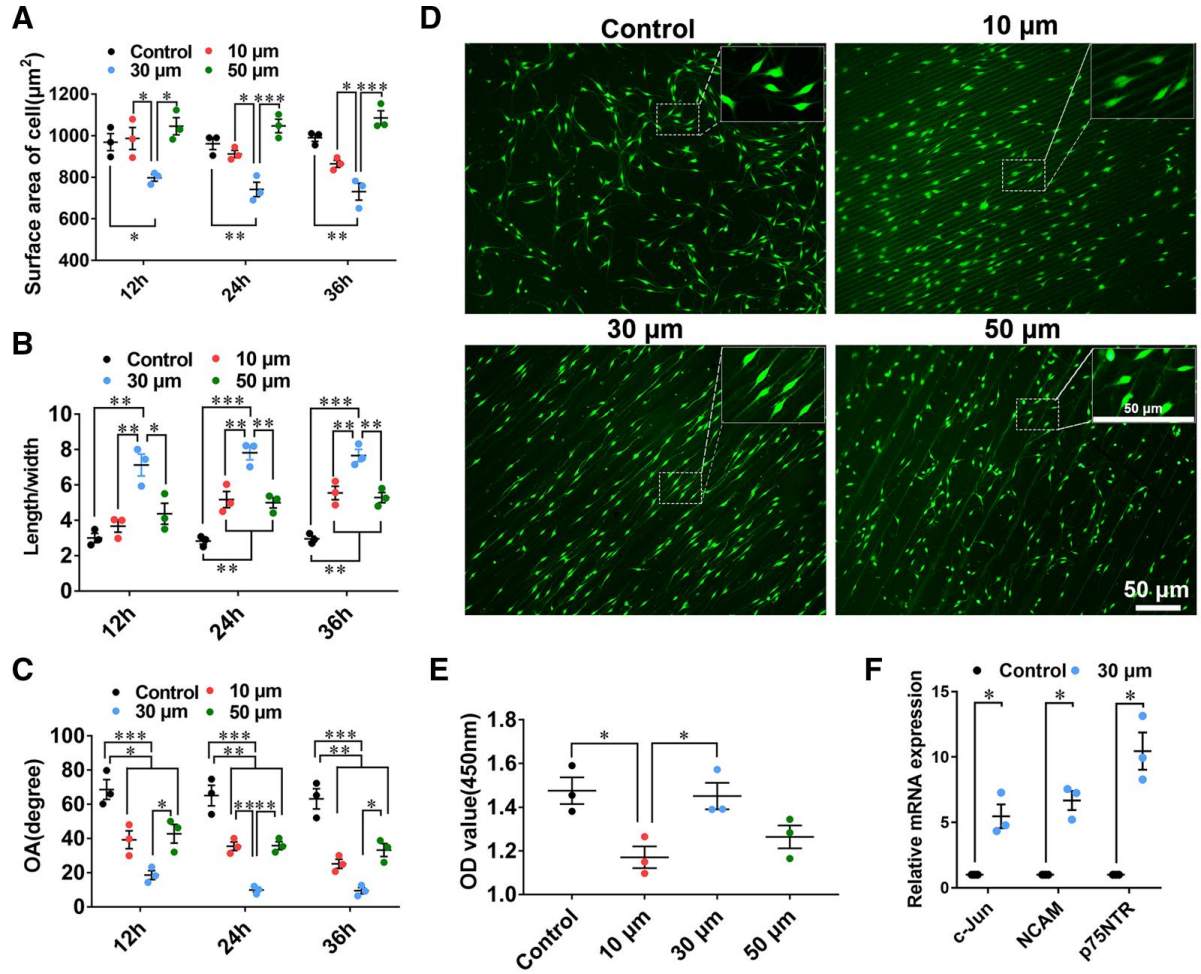
Morphological indexes and proliferation of SKP-SCs on different film surface. (**A**) Cell area, (**B**) cell aspect ratio and (**C**) cell angle of SKP-SCs was statistically analysed and showed significant improvement in micropatterned chitosan film groups especially in ‘30 μm’ surface group, with smaller cell area, more cell aspect ratio, and narrower cell angle, comparing to ‘flat’ group. (**D**) Representative fluorescence microscope images showed SKP-SCs morphology after culture on different surface samples for 36 h. The rectangle box at top-right was the magnification of the typical field of images. Scale bar, 50 μm. (**E**) The CCK-8 cell counting assay showed better proliferation status of SKP-SCs on ‘30 μm’ surface group and ‘flat’ surface group than that on ‘10 μm’ surface group, and no significant difference when compare ‘50 μm’ group with other groups. (**F**) The SKP-SCs from ‘30 μm’ surface group was further detected via qRT-PCR, showing increased mRNA expression level of repair-type Schwann cell phenotype markers c-Jun, NCAM and p75NTR, comparing to cells in ‘flat’ surface group. *n* = 3, *P <* *0.05, **P <* *0.01, ***P <* *0.001.

After culture for 36 h, the representative images showed GFP-positive SKP-SCs adhered on micropatterned film samples with preferable arrangement than that on flat film. The morphological indexes data of SKP-SCs on different samples at 12 h, 24 h and 36 h were showed in scatter-plots, respectively; compared with ‘10 μm’, ‘50 μm’ and ‘flat’ group, in ‘30 μm’ group the cell area was obviously decreased, the cell aspect ratio was significantly increased, and the cell angle was remarkedly decreased (Figure 3A–C). The representative image from different group showed SKP-SCs overall distribution morphology at low magnification and typical arrangement at high magnification (Figure 3D). Results demonstrated that lower cell area, longer elongation extent, and smaller cell angle of SKP-SCs was available via proper topographic cues induction.

### Cell proliferation and repair-type SC associated genes expression of SKP-SCs

CCK-8 assay results indicated that, after culture of 36 h the number of SKP-SCs in ‘30 μm’ group and ‘flat group’ was significantly more than that in ‘10 μm’ group, although there was no significant difference when SKP-SCs in ‘30 μm’ group compared with that in ‘flat’ group and ‘50 μm’ group (Figure 3E). Thus, the proliferative status of SKP-SCs was presented best in ‘30 μm’ group. On this basis, ‘30 μm’ group was selected to supply samples for the further detection of SKP-SCs mRNA expression and sequent experiments according to the safety consideration.

The qRT-PCR results demonstrated that the up-regulated gene expression of several commonly used repair-type SC markers c-Jun, neural cell adhesion molecule (NCAM), and neurotrophin receptor p75 (p75NTR) in SKP-SCs on ‘30 μm’ surface (Figure 3F). Our findings suggested that the SKP-SCs could be polarized by 30 μm micro-groove pattern induction effect.

### Promoted production of a panel of pro-neuroregenerative cytokines of SKP-SCs

**Figure 4.**
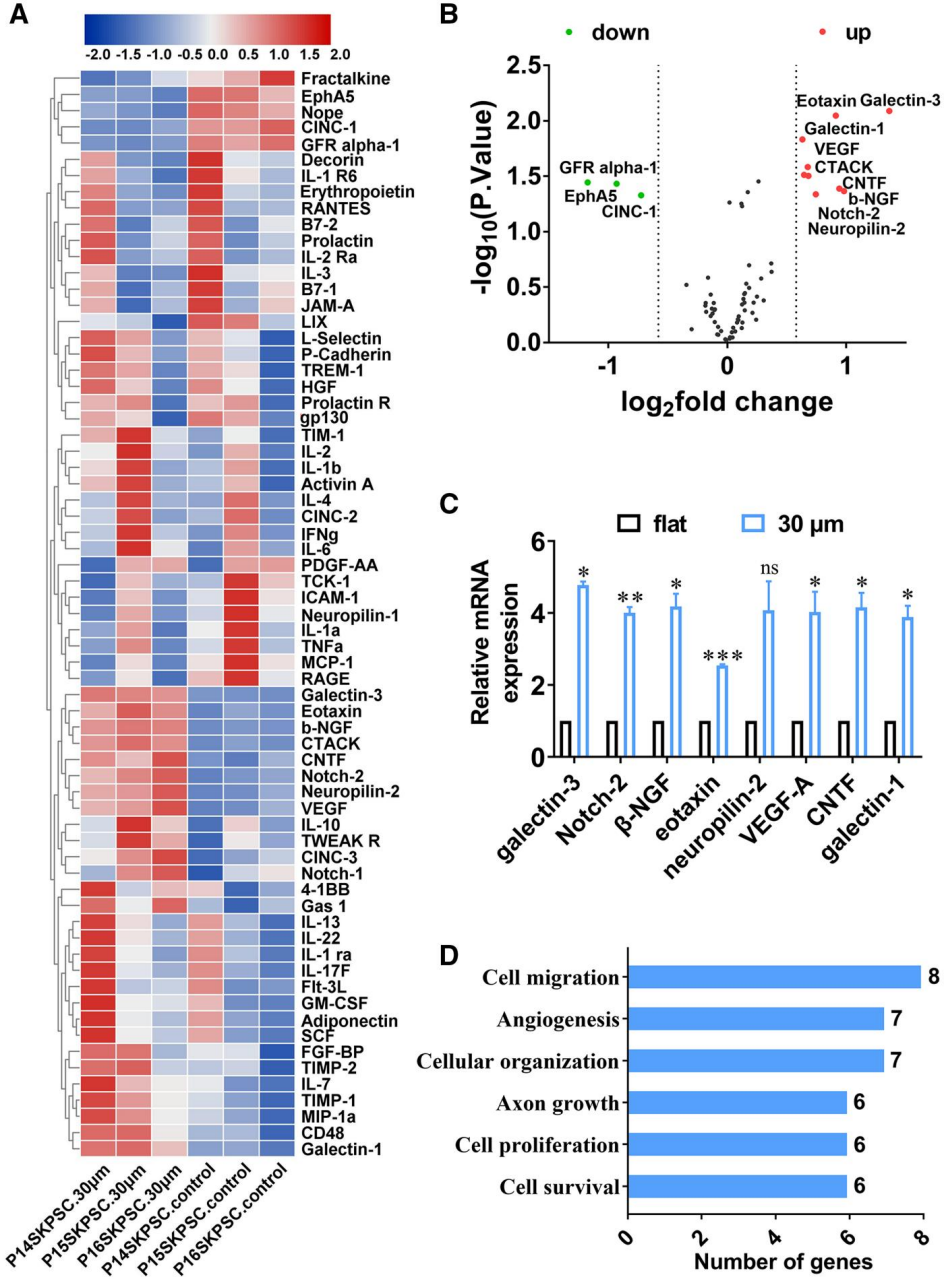
Detection and informative analysis of cytokines in SKP-SC-secretome. (**A**) Heat-map displayed the expression difference of 67 cytokines in SKP-SC-secretome from the ‘30 μm’ and ‘flat’ surface groups by cytokine array assay. (**B**) Volcano plot showing nine significantly up-regulated and three down-regulated cytokines in ‘30 μm-CM’ group compared with ‘flat-CM’ group according to log_2_FC > 0.58 and −log_10_ (*P*-value) > 0.89. (**C**) Nine up-regulated cytokines were subjected to further validation by qRT-PCR, results showed consistent expression except for CTACK, eight cytokines up-regulated in SKP-SCs from ‘30 μm’ surface group compared with that from ‘flat’ surface group, including galectin-3, eotaxin, Notch-2, neuropilin-2, galectin-1, CNTF, VEGF-A and β-NGF. (**D**) Histograms showed distinct pro-neuroregeneration-associated processes involved with the eight up-regulated cytokines via GO clusters analysis, with different functional terms on the ordinate and the number of genes within each cluster on the abscissa. *n* = 3, *P <* *0.05, **P <* *0.01, ***P <* *0.001.

According to the introduction of the cytokine GS67 array assay, it was designed to focus on rat chemotaxis cytokines and trophic factors. The heat map result showed the expression differences of 67 cytokines between the ‘30 μm-CM’ and ‘flat-CM’ group (Figure 4A). Although it seems the variation between three samples is large, among these cytokines, 12 differentially expressed cytokines were screened out according to log_2_fold change (log_2_FC)>0.58 and −log_10_ (*P*-value)>0.89, including nine up-regulated and three down-regulated cytokines that was illustrated in the volcano plot (Figure 4B). Hence, the up-regulated nine cytokines in ‘30 μm-CM’ were further verified by qRT-PCR. The data showed that eight cytokines, except CTACK (namely chemokine Ccl27), including galectin-3, eotaxin (gene name: Ccl11), Notch-2, neuropilin-2, galectin-1, ciliary neurotrophic factor (CNTF), vascular endothelial growth factor-A (VEGF-A), and beta-nerve growth factor (β-NGF), displayed consistent up-expression at gene level with the result of cytokine array assay (Figure 4C). The detailed data of array and qRT-PCR assay was showed in Supplementary Table S2. In order to connote the biological significance of the upregulated paracrine cytokines from ‘30 μm-CM’, the eight cytokines were categorized based on the biological functions predicted by the gene ontology (GO) cluster analysis, indicating these cytokines mainly involved in following pro-neuroregenerative biological processes: positive regulation of cell migration (eight genes), positive regulation of angiogenesis (seven genes), cytoskeleton organization (seven genes), axon growth including extension and neuron development (six genes), positive regulation of cell proliferation (six genes), cell survival such as negative regulation of apoptotic process and positive regulation of extracellular signal-regulated kinase 1/2 (ERK1/2) cascade (six genes) (Figure 4D).

### SKP-SC-secretome from ‘30 μm’ group promoted primary SCs proliferation and migration

**Figure 5.**
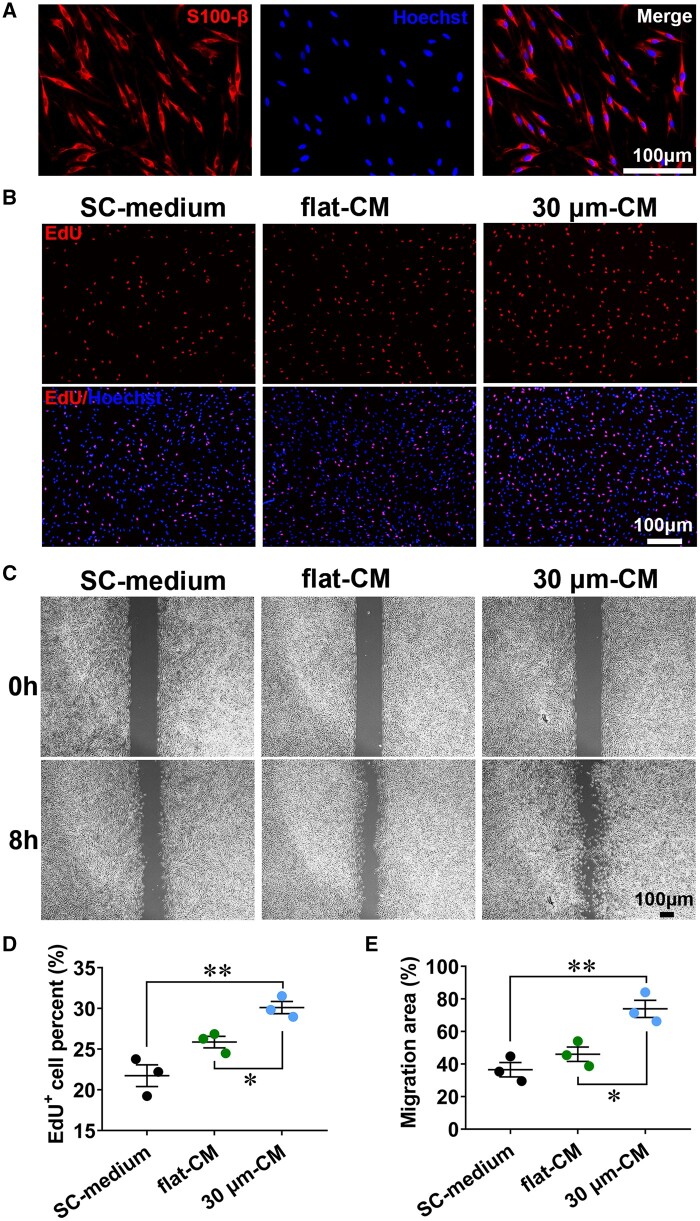
The SKP-SC-secretome from ‘30 μm’ surface group promoted primary SCs proliferation and migration. (**A**) Primary SCs showed positive expression of SC marker S100-β (red) with Hoechst labeled nuclei (blue). scale bar, 100 μm. (**B**) Representative images of EdU (red) labeled nuclei with Hoechst labeled nuclei (blue) background of SCs treated with ‘30 μm-CM’, ‘flat-CM’, and ‘MN-medium’ for 24 h, respectively. Scale bar, 100 μm. (**C**) Representative images of SCs scratch healing showed different migration area ratio of SCs in three groups after treatment for 8 h. Scale bar, 100 μm. (**D**) Statistical analysis showing the percentage of EdU positive SCs in ‘30 μm-CM’ group were remarkedly more than that in ‘flat-CM’ and ‘MN-medium’ group. (**E**) Statistical analysis showing the wound healing percentage of SCs in ‘30 μm-CM’ group were significantly higher than that in ‘flat-CM’ and ‘MN-medium’ group. *n* = 3, *P <* *0.05, **P <* *0.01.

As the result, the primary SCs displayed classic long-spindle morphology with side-by-side alignment, and positively expressed SC-specific marker S100-β with not less than 90% ratio (Figure 5A). To determine the effect of the SKP-SC-secretome on SCs growth, not only SKP-SC-secretome from ‘30 μm’ group and ‘flat’ group, but also ‘SC-medium’ were used to treat SCs. Results showed that, the ‘30 μm-CM’ could significantly increase the EdU-positive SCs ratio than ‘flat-CM’ and ‘SC-medium’ (Figure 5B and D), similarly, SCs migration area percentage was significantly larger in ‘30 μm-CM’ group than the other two groups (Figure 5C and E). Our findings demonstrated that SKP-SC-secretome from ‘30 μm’ group exhibited the significant pro-proliferation and pro-migration potency to primary SCs.

### SKP-SC-secretome from ‘30 μm’ group promoted neurite growth of intact MNs

**Figure 6.**
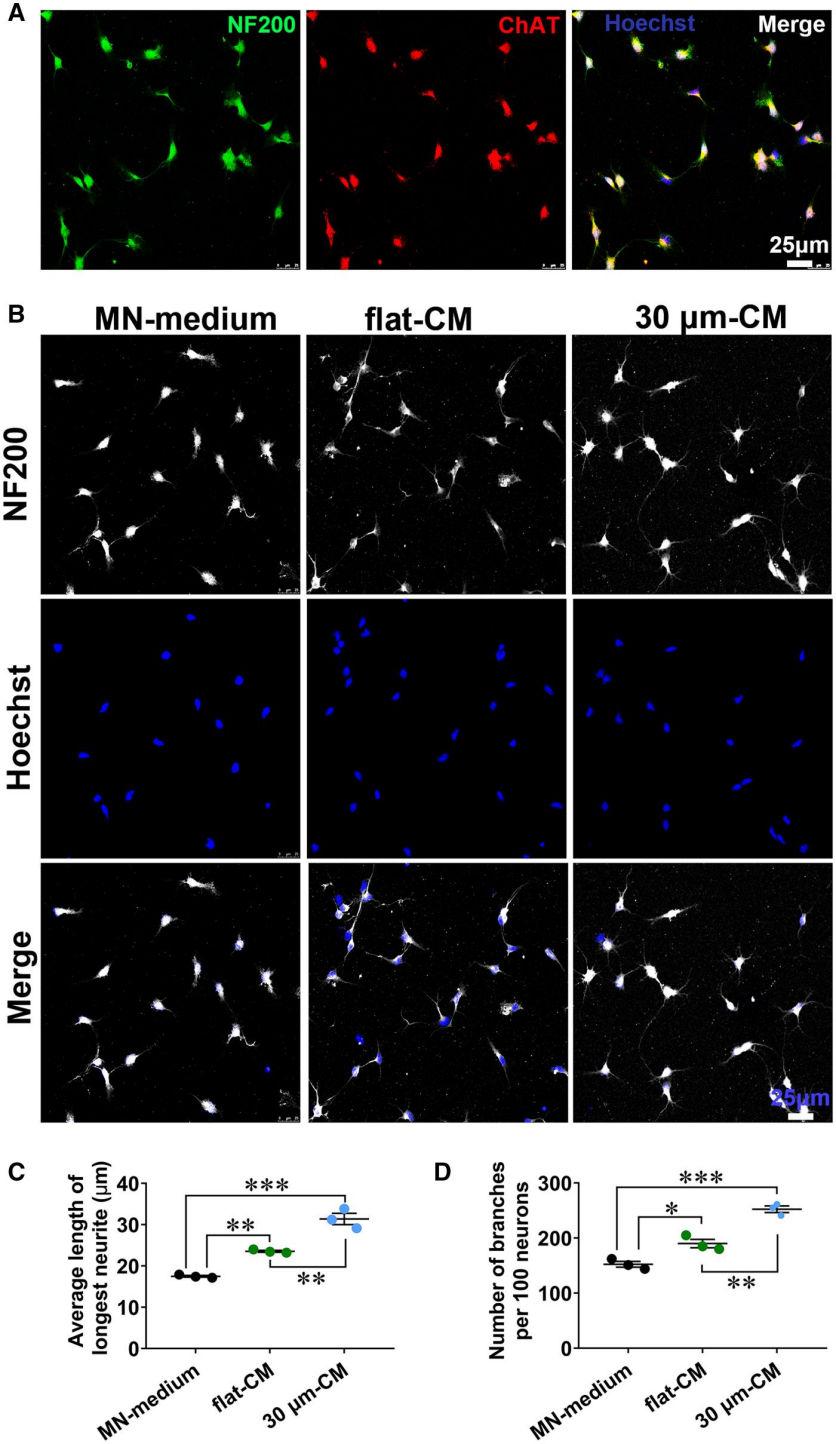
The SKP-SC-secretome from ‘30 μm’ surface group promoted MNs neurite growth. (**A**) Primary cultured MNs positively expressed NF200 (green) and ChAT (red) with Hoechst (blue) labeled nuclei. Scale bar, 25 μm. (**B**) Representative images of MNs showing better neurite growth of MNs in ‘30 μm-CM’ group than that in ‘flat-CM’ and ‘MN-medium’ groups after incubation for 24 h. Scale bar, 25 μm. (**C, D**) Statistical analysis showing the average length of the longest neurite and the number of branches per hundred neurons of MNs in ‘30 μm-CM’ group and ‘flat-CM’ group was increased compared with that in ‘MN-medium’ group, and the promoted growth was more significant in ‘30 μm-CM’ group than that in ‘flat-CM’ group. *n* = 3, *P <* *0.05, **P <* *0.01, ***P <* *0.001.

Results showed that primary MNs positively co-expressed NF200 and ChAT (a MN specific marker) with not less than 90% ratio (Figure 6A). The representative NF200 immunostaining images of MNs presented the neurites and branches of MNs after treatment with ‘30 μm-CM’ and ‘flat-CM’ and ‘MN-medium’, respectively (Figure 6B). The average length of the longest neurite and the branches number of per hundred neurons were remarkedly promoted by ‘30 μm-CM’ and ‘flat-CM’ treatment compared with ‘MN-medium’ treatment, moreover, the enhancement effect was better in ‘30 μm-CM’ group than that in ‘flat-CM’ group (Figure 6C and D).

### SKP-SC-secretome from ‘30 μm’ group promoted axonal regrowth of axotomized MNs

**Figure 7.**
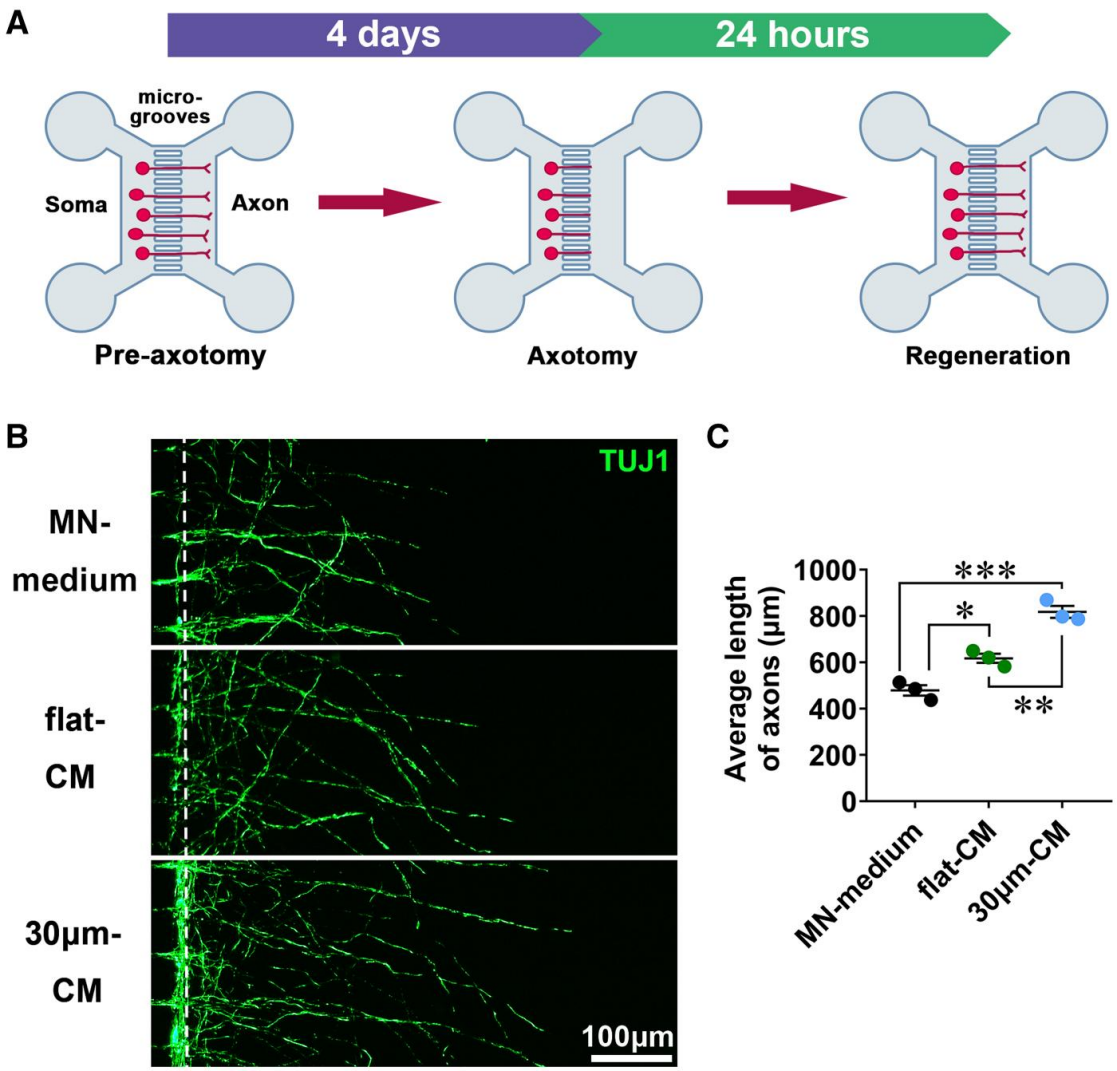
The SKP-SC-secretome from ‘30 μm’ surface group promoted MNs axonal regrowth. (**A**) Schematic of axonal outgrowth from MNs soma chamber to axon chamber through grooves for 4 days, followed by the axotomy of the sprouting axons and axonal regrowth for 24 h. (**B**) Representative images of MNs showed different length of regenerated axons of damaged MNs after treatment with ‘30 μm-CM’, ‘flat-CM’, and ‘MN-medium’, respectively. Scale bar, 100 μm. (**C**) Statistical analysis showed that the average length of MNs regenerated axons in ‘30 μm-CM’ group and ‘flat-CM’ group was longer than that in ‘MN-medium’ group, and the promoted axonal regrowth was more significant in ‘30 μm-CM’ group than that in ‘flat-CM’ group. *n* = 3, *P <* *0.05, **P <* *0.01, ***P <* *0.001.

The schematic showed that in a microfluidic device the axonal outgrowth traversed through the microgrooves at 4 days after MNs culture in soma chamber, followed by axotomy of the sprouting axons, axons were permitted to regrow for 24 h (Figure 7A). Representative immunostaining images of MNs with regenerated axons in three groups were presented, respectively (Figure 7B). After treatment for axotomy-injured MNs, ‘30 μm-CM’ and ‘flat-CM’ significantly promoted the average length of regenerated axons of MNs compared with ‘MN-medium’, and axons in ‘30 μm-CM’ group were longer than that in ‘flat-CM’ group statistically (Figure 7C).

## Discussion

Recently, the progress in neural tissue engineering research have indicated that constructing appropriate biomaterial scaffold to combine with seed cells is always a hot topic in regenerative medicine development process. Natural biomaterials act as instructive engineered microenvironments, that can effectively tune cellular morphology and function in neural tissue engineering [39]. To guide and align the cell growth in the desired direction, scaffolds that closely mimic the native ECM environment would be able to incorporate different biomimetic characteristics at a molecular level from a perspective on the structural requirements [40]. Firstly, the structural requirements need to be addressed, the structural design considerations for scaffolds in the peripheral nervous system and spinal cord is summarized, including reproducing the features of the natural ECM, the alignment of the scaffolds, surface topography.

Our previous works have reported that the native rat SCs once cultured on anisotropic topographic chitosan membrane with micro-grooves, would show enhanced cellular viability and oriented alignment than that on flat film [23], as well as strengthened reparative function associated with transformed gene phenotype [34]. The improved SC response was due to the increased asymmetry of micro-grooved chitosan membrane [41]. Engineering the microtopography of biomaterial substrates is a feasible way to control the cell behaviors by mimicking the natural contact-mediated signaling events [42]. Although the encouraging progress for nerve regeneration is bringing hope to PNI and SCI patients, notably, native SCs had to be gradually replaced by stem cell/progenitors and derived Schwann-like cells due to the insufficient source [43].

In order to obtain the practical efficacy, the potential of Schwann-like cells and the biocompatibility of chitosan were matched to construct an achievable combination. The topographical modification of the chitosan film, including the alterations to the types, sizes and spacing of surface patterns, can be an approach to guide intrinsic signaling pathways and control neural cell behaviors [23]. Here we provided evidences to verify the feasibility of the combination of SKP-SCs with the micropatterned chitosan scaffold, including the cellular arrangement organization and paracrine function optimization of SKP-SCs. The imposed topography of micro-grooved chitosan film surface can effectively provide contact guidance for the adhesion and spreading, alignment and orientation, cytokine secretion and underlying genic phenotype transition of SKP-SCs.

We obtained similar interplay by using SKP-SCs as substitute of native SCs. Especially among different surface groups, SKP-SCs on ‘30 μm’ surface showed optimum status. Although surface micropatterning is a strong physical tool for modulating cell behavior, responses to micropatterns are highly dependent on the cell type [44]. Encouragingly, the morphology indexes of SKP-SCs demonstrated that reshaped Schwann-like cells were in response to the micropatterned chitosan surface, grew more orientationally along the microgrooves with 30 μm width than along with other width grooves, that meant the matched pattern size approached to the cellular size of rat SKP-SCs was about 30 μm, otherwise, cells randomly spread on the flat surface or laid across the narrowed microgrooves. Similarly, previous work indicated the proper microgroove size of chitosan film for primarily cultured rat SCs was about 20–30 μm [34].

Additionally, it was well known that in early stage of nerve regeneration *in vivo*, native SCs present longitudinally oriented Büngner bands. Here the arrangement adaptation of SKP-SCs resembled the behavior, that facilitated to the direction guidance for subsequent axon extension. Previously, researchers also found that tissue engineered bands of Büngner formed by rat primary SCs or human gingiva-derived SCs could both accelerate motor and sensory axonal outgrowth [45], that simulated the environmental topography signals coming from the disrupted area in the natural healing process of peripheral nerves [46]. On the basis, once rolling the anisotropic chitosan film up to fabricate conduits with topographic inner wall, the conduits can be further administrated on long-distance nerve defect and segmental SCI, importantly, the survival and arrangement of the encapsulated SKP-SCs would be guaranteed [22, 47]. Taken together, here chitosan scaffold resembled the native tissue excreted ECM, can confer effective contact guiding for the arrangement and growth direction of seeded SKP-SCs via physiochemical signals. SKP-SCs behaved similar as native SCs, thereby were again confirmed as promising Schwann-like cells.

In agreement with the morphological phenotype transition, the genic phenotype change of SKP-SCs induced by ‘30 μm’ surface was also verified similar to repair-type SCs. Here induced SKP-SCs with best arrangement on ‘30 μm’ anisotropic chitosan film surface was selected to detect the expression of repair-type SC gene markers, such as c-Jun, NCAM and p75NTR [48, 49], that were all significantly elevated. Afterwards, we further confirmed the paracrine function optimization of the repair-phenotype SKP-SCs. SKP-SC-secretome from ‘30 μm’ surface group were harvested and utilized. Both the migration and proliferation of native SCs and the neurite growth of native MNs were enhanced after the ‘30 μm-CM’ treatment. Subsequently, ‘30 μm-CM’ was administrated to prove the reparative effect of induced SKP-SC-secretome on the regrowth of axons by the microfluidic axotomy-injury model of MNs. Notably, the derived secretome presented significantly beneficial effect on native SCs and MNs growth, that partially depending on a panel of up-regulated paracrine cytokines. Thus, a panel of increased paracrine cytokines at protein secretion level and mRNA expression level suggested that the induced SKP-SCs presented the phenotype transition of paracrine function in aiding neural regeneration. Which further revealed that the response of SKP-SCs to anisotropic topography surface was profit to neural regeneration, and enable future translational application research, even implying optional strategy from engineered cell therapy to cell-free therapy [43].

Furtherly, the informative cluster analysis of the eight up-regulated cytokines, including galectin-3, eotaxin (Ccl11), Notch-2, neuropilin-2, galectin-1, CNTF, VEGF-A, and β-NGF, demonstrated their relevance to multiple neural regenerative function. All eight cytokines were clustered involved in positive regulation of angiogenesis, consistently, researchers previously have reported that Schwann-like cell-sourced CM could promote angiogenesis and nerve regeneration [50]. Previously researchers ever reported other up-regulated pro-regeneration associated gene markers in native SCs cultivated on anisotropic chitosan film surface, such as smad 6 (neuronal plasticity and axon regeneration), and β-actin (cytoskeleton development) [34]. Besides, the concerned NGF also has been reported could be promotively secreted by native SCs cultivated on micro-grooved chitosan film surface [23, 35], other types of micropatterns were also well documented could promote SCs secrete NGF for axonal regeneration [51, 52]. These factors associated with repair phenotype of SCs would potentialize the pro-regenerative function of engineered SCs or Schwann-like cells in cell transplantation microenvironment [53]. Our cytokines detection and analysis similarly demonstrated the up-expression of the pro-regeneration relevant gene markers in surface topographic cues induced SKP-SCs, enabled to promote cellular organization and axonal growth. The promotive effect of these up-regulated paracrine cytokines were also manifested in several other aspects, cell survival, cell proliferation, and cell migration that were closely associated with ERK1/2 cascade signal pathway, which has been reported implicated with c-Jun, the classic repair-type SC marker [54]. It was implied that the genic phenotype transition acted as intracellular response was the underlying regulation mechanism of the paracrine function phenotype.

In the present study, although the SKP-SCs exhibited multiple aspects of response facilitating to neural regeneration, the interactions between anisotropic topography and adhered SKP-SCs still require further exploration. For instance, whether the other contributors such as ECM components and EVs in SKP-SC-secretome might be influenced by topography contact guiding. Since ECM from SKP-SCs was applied as the acellular scaffold constructs conduit [55], a pro-angiogenesis nerve graft constructed by SKP-SC ECM has been newly reported [33]. Actually, the EVs isolated from regular SKP-SC-secretome has been applied in our previous experiments to repair damaged neurons and rat defective nerve successively [32, 56, 57]. Moreover, the undetected cytokines in SKP-SC-secretome with more other possible effects still remain unclear, such as anti-inflammation, alleviating fibrous dense scarring formation of PNI axon growth, rejecting the growth inhibitor in SCI scar etc. As that, the *in vivo* efficacy and other underlying mechanisms were also worthy to be further investigated. In future work, high throughput proteomics detection and analysis for SKP-SCs secretome would be considered.

Meanwhile, given the complexity associated with neural regeneration, the innovation in the process and technology of preparing biomaterial scaffolds, such as 3D printing and electrospinning technology, has provided enormous potential for the transformational application of tissue engineering nerves [58]. For advanced neural regeneration, an approach to the design and manufacturing of 3D printed neural devices could provide next-generation opportunities, through the production of anatomically accurate geometries, spatial distributions of cellular components, and the incorporation of therapeutic biomolecules [59]. These considerations would be concerned in future work.

## Conclusion

Our study utilized surface topography of chitosan scaffold to induce Schwann-like cells. The micropattern act as a vital physical property can strongly impact the biological environment of surrounding cells and tissues. It was demonstrated that appropriate topographic contact guidance cues stimulation successfully induced the beneficial response of SKP-SCs, including advantageous cellular organization orientation, genic repair phenotype transition, and augmented paracrine potency. Furthermore, a panel of up-regulated cytokines were highlighted in SKP-SC-secretome from ‘30 μm’ group, and these cytokines were revealed closely relative to neural regeneration via cluster analysis. Thereby the combined chitosan substrate and Schwann-like cells would exhibit promotive neural regeneration action synergistically via creating a beneficial physicochemical microenvironment. Looking forward, these findings can provide inspiration in the design of clinical scaffolds as potential replacements for the autografts, be used for bridging the lesion site of neural tissue. Collectively, SKP-SCs could conquer the barrier of lack of native SCs, the combination of Schwann-like cells with anisotropic chitosan scaffold would provide novel insights into the further application research work.

## Supplementary Material

rbae005_Supplementary_Data
