# Supplementary material for: Anisotropic microtopography surface of chitosan scaffold regulating skin precursor-derived Schwann cells towards repair phenotype promotes neural regeneration
Source: Regen Biomater. 2024 Jan 27;11:rbae005. doi: 10.1093/rb/rbae005 (PMC10898340; doi:10.1093/rb/rbae005)
Supplement: rbae005_Supplementary_Data [file rbae005_supplementary_data.zip › Table S1 mRNA primers.doc]

| Gene name | Forward primer (5' to >3') | Reverse primer (5' to >3') |
| --- | --- | --- |
| *c-Jun* | TGTCTGTATGCTGGGGTGA | GGTTGCTGGGGAGAGAGA |
| *NCAM* | CAAGTTCTGTGGGAAGGC | CTGGGATGTGGGAGGTT |
| *p75NTR* | GACAGTGCAACGCTTGATG | GAATGTGGGACAGGACAGG |
| *Galectin-3* | GGTTGCGGTCAATGATGTT | CGCTGGTGAGGGTTATGTC |
| *CTACK* | AGGCAACCACTCCCAACCAG | GGCTCGGGCACATTTTCTTT |
| *Eotaxin* | CTCACGGCCACTTCCTTCAC | TTTTCTTGGGGTCAGCACAG |
| *Notch-2* | CCATTGTCCCAACCTATCACC | CAGCATTTGAGGAGGCATAAC |
| *Neuropilin-2* | TCAGATCGTATTCGAGGGAGT | AGTGGGACATCGGTGCTTAT |
| *Galectin-1* | ACCTGTGCCTACACTTCAACC | CCCATCGTCCTTGCTGTTAC |
| *TIM-1* | TTGGACCTACTTGAGTGTTACGA | CATCACCACATCCGCCTTCT |
| *CNTF* | ATGACTGAGGCAGAGCGACT | AAAGGCAGAAACTTGGAGCA |
| *VEGFα* | CCACGACAGAAGGGGAGCA | ACACCGCATTAGGGGCACA |
| *βNGF* | CCCGCAACATCACTGTGGAC | GGTGGAGGCTGGGTGCTAAA |
| *GAPDH* | GGCTCTCTGCTCCTCCCTGT | CGTTCACACCGACCTTCACC |

Table S1 mRNA Primers
