# Supplementary material for: Anisotropic microtopography surface of chitosan scaffold regulating skin precursor-derived Schwann cells towards repair phenotype promotes neural regeneration
Source: Regen Biomater. 2024 Jan 27;11:rbae005. doi: 10.1093/rb/rbae005 (PMC10898340; doi:10.1093/rb/rbae005)
Supplement: rbae005_Supplementary_Data [file rbae005_supplementary_data.zip › Table S2 Relative mRNA expression of up-regulated cytokines by SKP-SCs.doc]

| Protein ID | *P*.Value  (Array) | Fold change (Array) | Fold change (qRT-PCR)  (mean±SEM) |
| --- | --- | --- | --- |
| galectin-3 | 0.008 | 2.572 | 4.777±0.3556 |
| Notch-2 | 0.043 | 1.974 | 4.006±0.1589 |
| β-NGF | 0.046 | 1.921 | 4.182±0.3556 |
| eotaxin | 0.010 | 1.882 | 2.542±0.03366 |
| neuropilin-2 | 0.046 | 1.675 | 4.079±0.8021 |
| VEGF-A | 0.031 | 1.605 | 4.027±0.5651 |
| CTACK | 0.026 | 1.598 | 1.042±0.09829 |
| CNTF | 0.031 | 1.565 | 4.159±0.4056 |
| galectin-1 | 0.015 | 1.550 | 3.888±0.3135 |

Table S2 Relative mRNA expression of up-regulated cytokines by SKP-SCs
